# Analysing the meta-interaction between pathways by gene set topological impact analysis

**DOI:** 10.1186/s12864-020-07148-y

**Published:** 2020-10-27

**Authors:** Shen Yan, Xu Chi, Xiao Chang, Mengliang Tian

**Affiliations:** 1 College of Agronomy, Sichuan Agricultural University, Chengdu, 611130 Sichuan China; 2 Beijing Institute of Genomics, Chinese Academy of Sciences, Beijing, 101300 China; 3 China National Center for Bioinformation, Chaoyang, Beijing, 101300 China; 4 Department of Dermatology and Venereal Disease, Xuanwu Hospital, Capital Medical University, Beijing, 100053 China

**Keywords:** Topological pathway analysis, Algorithm development, Functional module

## Abstract

**Background:**

Pathway analysis is widely applied in transcriptome analysis. Given certain transcriptomic changes, current pathway analysis tools tend to search for the most impacted pathways, which provides insight into underlying biological mechanisms. Further refining of the enriched pathways and extracting functional modules by “crosstalk” analysis have been proposed. However, the upstream/downstream relationships between the modules, which may provide extra biological insights such as the coordination of different functional modules and the signal transduction flow have been ignored.

**Results:**

To quantitatively analyse the upstream/downstream relationships between functional modules, we developed a novel GEne Set Topological Impact Analysis (GESTIA), which could be used to assemble the enriched pathways and functional modules into a super-module with a topological structure. We showed the advantages of this analysis in the exploration of extra biological insight in addition to the individual enriched pathways and functional modules.

**Conclusions:**

GESTIA can be applied to a broad range of pathway/module analysis result. We hope that GESTIA may help researchers to get one additional step closer to understanding the molecular mechanism from the pathway/module analysis results.

**Supplementary information:**

**Supplementary information** accompanies this paper at 10.1186/s12864-020-07148-y.

## Background

Pathway analysis is a routine process in transcriptome analysis used to gain biological insights. As reviewed in several recent works [1–3], such analysis can be roughly categorized into three groups: Over Representative Analysis (ORA), Functional Class Scoring (FCS), and Pathway Topology Based (PTB) analysis. The ORA methods, exemplified by GO enrichment analysis [4, 5], are based on hypergeometric distribution and the over-representation of the shared genes between pathways. The FCS method, exemplified by GSEA [6], incorporates the level of gene expression changes in weighting the calculation of the enrichment score. Since these tools do not consider the topological structure of the gene interaction networks, more recently developed algorithms convert the topological structure of the pathways into impact scores (e.g. SPIA) [7] or weights (e.g. CePa, NetPathMiner) [8, 9] before incorporating them in the enrichment algorithm.

Despite the wide application of these tools in transcriptome analysis, there are biases and false positives in these pathway analysis results. Donato et al. 2013 [10] showed that unrelated pathways may also be significantly enriched by pathway analysis due to shared genes (crosstalk) in the pathways. They designed a “crosstalk analysis” to identify such crosstalk effects and remove them from the pathway analysis results, while generating new sub-pathways that represent functional modules. Although the refinement of the enriched pathways and the extraction of functional modules greatly improved the rationality of the results, the interpretation of the results is based upon the prior knowledge of the pathways and modules, which provides limited evidence to distinguish the true positive from false positive if a seemingly unrelated pathway/module is significantly enriched.

To explore the biological insights beyond individual enriched pathways and functional modules and generate extra clues to help inferring the molecular mechanisms, we propose a new strategy that quantitatively assesses the upstream/downstream relationships between pathways/modules. Our new algorithm uses topology, but also utilizes gene-gene interaction at supra-pathway level. This is based on the idea that the pathways/modules of a biological process are coordinately regulated, hence they may have interactions, especially upstream/downstream relationships. To our knowledge, there is currently no such algorithm/software to identify the upstream/downstream relationships between pathways/modules. The existing pathway crosstalk analysis software (e.g. CrossTalkZ and SPATIAL) [11, 12] investigates the interactions between pathways to search for more shared features of the pathways than solely shared genes. As a result, these algorithms are able to reduce the false negative rate, but not the false positive rate, of the pathway analysis results. Additionally, along with other crosstalk analysis software such as BinoX [13] and FoPA [14], they are unable to assess the upstream/downstream relationships..

In this study, we developed a novel algorithm called GEne Set Topological Impact Analysis (GESTIA). GESTIA is based on a global network merged from KEGG pathways [15, 16]. Based on the network, GESTIA can take user-defined gene sets/functional modules or precompiled pathways and compute the relative influence score of one pathway/module on the other. A positive GESTIA score indicates the former pathway/module is upstream of the latter, and vice versa. We demonstrated that GESTIA score does not directly correlate with the similarities or the functional correlations of the pathways/modules, which means that even though two pathways/modules may share no common genes, they can still have strong GESTIA scores, indicating that one pathway/module acts upstream of the other and, therefore, has a strong impact on that other pathway/module but not vice versa. On the other hand, pathways/modules that share a large number of genes might not have a high GESTIA score, since the two pathways/modules may only interact with each other mutually. We applied GESTIA scores to analyze the relative influence of the DNA repair pathways and oncogenic pathways on each other, and showed that GESTIA score can be a good indicator of the upstream/downstream relationships of the pathways. Additionally, we used GESTIA on published transcriptome datasets and demonstrated the practicality of using GESTIA scores to infer molecular mechanisms from dispersed pathways/modules by assembling them into a topological structure. GESTIA has been implemented in R package (http://github.com/yanshen2953/GESTIA).

## Results

### Development of the GEne set topological impact analysis (GESTIA)

**Fig. 1:**
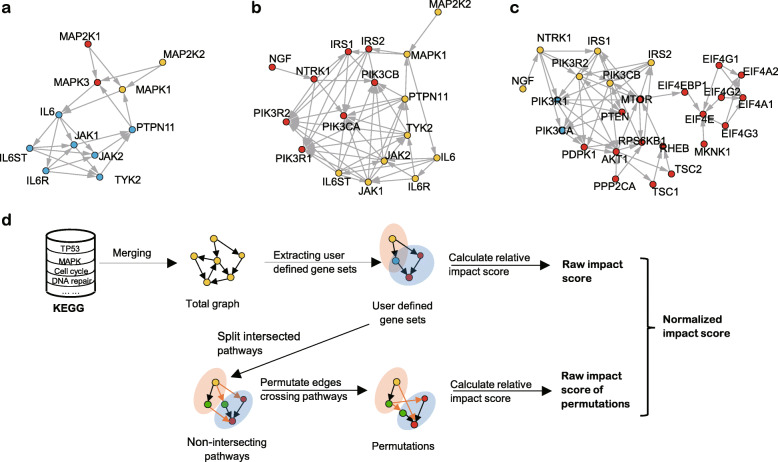
Representative pathway interactions and the design of GESTIA. **a.** Visualization of the gene interactions between REACTOME_MAPK1_ERK2_ ACTIVATION and REACTOME_MAPK3_ERK1_ACTIVATION. Vertices in red represent genes in the first of the two pathways, yellow represent genes in the second, and blue represent shared genes. **b.** Visualization of the gene interactions between REACTOME_MAPK1_ERK2_ACTIVATION and REACTOME_PI3K_ AKT_ACTIVATION. The color set of the vertices is the same as in a. **c.** Visualization of the gene interactions between BIOCARTA_MTOR_PATHWAY and REACTOME_PI3K_ AKT_ACTIVATION. from MSigDB. The color set of the vertices is the same as in a. **d.** The design of GESTIA. The color set of the vertices is the same as in a, and green represent pseudo-vertices

To assess the relationships between gene sets or pathways, the significance of the enrichment of the overlapping genes is often used as an indicator. However, the number of overlapping genes between pathways does not reflect the upstream/downstream interactions of the pathways. As observed in the example signaling pathways (REACTOME_MAPK1_ERK2_ACTIVATION and REACTOME_MAPK3_ ERK1_ACTIVATION from Molecular Signature DataBase, MSigDB, Fig. 1a) [6, 17], it is possible for two pathways to share a high proportion of genes, yet exhibit nearly equal dominant effects on each other. The “dominant effects” here means a “tail to head” interaction of the upstream pathway to the downstream pathway. On the other hand, even if two pathways share few or even no common genes, they might still be closely correlated with each other. This is illustrated in Fig. 1b, which displays REACTOME_MAPK1_ERK2_ACTIVATION and REACTOME_PI3K_AKT_ ACTIVATION signaling pathways from MSigDB, and in Fig. 1c, which displays BIOCARTA_MTOR_PATHWAY and REACTOME_PI3K_AKT_ACTIVATION from MSigDB. The two pathways in Fig. 1a and Fig. 1b show an almost symmetrical topology, which is also true in the biological sense. The yellow pathway in Fig. 1c is clearly located upstream of the red pathway. In order to quantitatively assess the upstream/downstream relationships of two pathways/modules, especially the relationships exemplified by Fig. 1b and Fig. 1c, we developed a novel algorithm named “GEne Set Topological Impact Analysis” or GESTIA (Fig. 1d).

Since genes may have different functions or interactions in different pathways/modules, interrogating the interactions between pathways in the context of a global network enables comprehensive assessment of the gene interactions beyond the individual pathways being studied (see Fig. 1b as an example). Suppose Gene A and Gene B are from two different pathways, and they do not interact with each other according to these two pathways. However, they may interact with each other in a third pathway. This interaction will only be shown if we take the gene interactions from the third pathways into consideration when we study the relationship of the first two pathways. And this the reason that two pathways sharing no common genes are still possible to interact with each other. Considering the situations like this, we constructed a global network from the KEGG pathway database before calculating GESTIA scores. The code for merging and filtering the global network can be found in Additional file 3.

To quantitatively assess the upstream/downstream relationships of pathways/modules, we designed the GESTIA score to reflect the relative influence of one pathway/module on the other, since “upstream” usually implies that one pathway/module acts on another pathway/module. Therefore, we converted the calculation of GESTIA score into the comparison (subtraction) of the two calculable influence scores, pathway/module *A* on pathway/module *B* and the reverse, *B* on A. To calculate the influence score of *A* on *B*, we took the sum of all the influences of the genes in *A* on *B* as the raw influence score. The influence score of *B* on *A* was calculated similarly.

However, with any given topological structure of two pathways/modules, there is a chance for random interactions between genes that belong to different pathways/modules. The influence scores of these random interactions, therefore, form the null distribution. Because of the variability of the topological structure of pathways, it is difficult to construct a universal null distribution for all the combinations of different pathways. Therefore, we permutated the interactions between the two pathways/modules being studied to calculate an empirical null distribution of the influence score, while maintaining the internal topological structure of each of the two pathways/modules.

For pathways with overlapping genes, the interactions between the shared genes and other genes can be considered as both intra-pathway/module and inter-pathway/module interactions, which impede the permutations, because if we perturb these inter-pathway/module interactions, we will have to change the pathways’/modules’ topological structure, since these interactions are also intra-pathway/module. To overcome this dilemma, we create pseudo-vertices in the network that duplicate the shared genes (Fig. 1d). In this way, the two previously overlapping pathways/modules are split into two non-overlapping pathways/modules, which enables the same permutation strategy as for non-overlapping pathways/modules. At the same time, since this splitting process does not perturb the topological structure of individual pathways/modules, the raw influence scores remain the same. This overlap-splitting strategy successfully solved the difficulty of permutating pathways/modules with overlapping genes, enabling the unbiased modeling of the null distribution of the influence scores. Meanwhile, similar to GSEA [6], we estimated the significance of the relative influence scores by calculating the chance of randomly permutated interactions getting a more significant relative influence score than the real one.

### Measurement of the upstream/downstream position of two pathways by GESTIA score

We applied GESTIA first on the example pathway pairs in Fig. 1. We calculated a GESTIA score of 0.013 and a *p*-value of 0.49 for Fig. 1a, which is consistent with the demonstrated topological structure of the two parallel pathways (red and blue vertices && yellow and blue vertices). For Fig. 1b, we got a positive GESTIA score of 0.011, which indicated “slight” upstream activity of the pathway REACTOME_MAPK1_ERK2_ACTIVATION (yellow vertices) to the pathway REACTOME_PI3K_AKT_ ACTIVATION (red vertices). Indeed, although all the arrows between the two pathways end in the REACTOME_PI3K_ AKT_ACTIVATION pathway, almost all of the endpoint genes (IRS1, IRS2 etc.) of these interactions are downstream of this pathway, meaning that if the interactions are randomly chosen between the two pathways while maintaining the directions, there is a large chance that the resulted random raw GESTIA score is higher than the real one, because the randomly chosen interactions are likely to end in more upstream genes of REACTOME_PI3K_AKT_ACTIVATION. Therefore, the significance of these upstream/downstream relationships is low, reflected by a *p*-value of 1.0. For Fig. 1c, pathway BIOCARTA_MTOR_PATHWAY (red and blue vertices) clearly sits downstream of pathway REACTOME_PI3K_AKT_ACTIVATION (yellow and blue vertices); therefore, the strong negative GESTIA score (− 1.4) and a small *p*-value of 0.001 is as expected. In general, GESTIA score and its *p*-value can correctly indicate upstream/downstream relationships between two pathways.

**Fig. 2:**
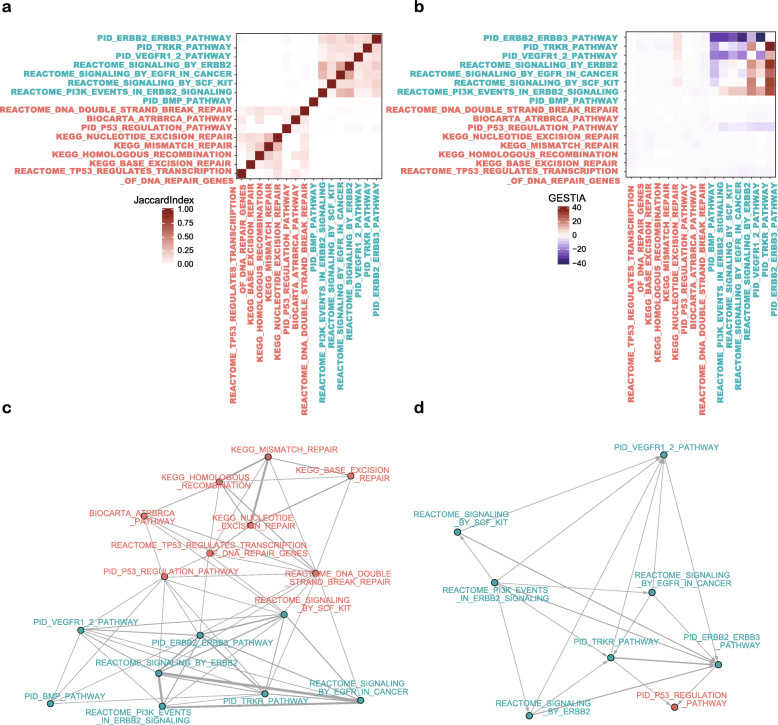
Demonstration of the similarities and upstream/downstream relationships between oncogenic pathways and DNA repair pathways. **a.** The heatmap of the Jaccard Index matrix of oncogenic pathways and DNA repair pathways. Fonts in blue represent oncogenic pathways, fonts in pink represent DNA repair pathways. **b.** The heatmap of the GESTIA scores between oncogenic pathways and DNA repair pathways. The color set and orders of the rows and columns are the same as in a. **c.** The undirected weighted network constructed using the Jaccard Index matrix as an adjacency matrix. The weights of the edges are proportional of the Jaccard Index scores in the matrix. Blue represent oncogenic pathways, pink represent DNA repair pathways. **d.** The directed weighted network constructed using the GESTIA matrix as an adjacency matrix. Negative values were filtered out because of the symmetry of the matrix. The color set are the same as in c

To compare GESTIA with commonly used pathway analysis tools, we applied GESTIA on the DNA repair pathways and oncogenic pathways, most of which were selected from Chi et al. 2019 [18]. We first calculated the matrix of the Jaccard Index, which is an indicator of similarity, for the oncogenic and DNA repair pathways (Fig. 2a, Additional file 4). The oncogenic pathways and the DNA repair pathways can be clearly separated into two groups by the number of shared genes between them. Using the Jaccard Index matrix as an adjacency matrix, we constructed an undirected weighted network of these pathways, which shows a similar tendency (Fig. 2c).

The heatmap of GESTIA score matrix, however, shows a distinct structure of relationships between these pathways (Fig. 2b, Additional file 5). Since the sign of the GESTIA score reflects the upstream/downstream relationship of the two pathways, we arranged the matrix to be the GESTIA scores of row pathways over column pathways; therefore, a positive score indicates that the row pathway is upstream of the column pathway. As expected, the DNA repair pathways shows fewer interactions because of the distinct mechanisms these pathways are involved in. On the other hand, all of the growth factor receptor pathways are upstream of PID_P53_REGULATION _PATHWAY, as expected. However, there are two special cases that showed unexpected relationships with other pathways. One is PID_ERBB2_ ERBB3_PATHWAY, which is downstream of all other oncogenic pathways. Examination of this pathway and its interactions with other pathways showed that, unlike other growth factor receptor pathway collections, this pathway contains more downstream pathway genes, e.g. genes from the PI3K/AKT and MAPK signaling pathways, as exemplified by the comparison of PID_ERBB2_ERBB3_PATHWAY and REACTOME_SIGNALING_BY_ERBB2 (Additional file 1). Although the names of these two pathways may create an impression that they should be similar, or at least partially similar because they are both about ERBB2’s pathway, the significant differences in the gene members of these two pathways suggest that the annotations and the components of the pathways might differ from database to database, hence researchers need to check the components of the pathway collections in detail to accurately interpret the enriched pathways. The other unexpected pathway is REACTOME_PI3K_EVENTS_IN_ERBB2_ SIGNALING, which is upstream of other growth factor receptor pathways. This is because it contains fewer downstream genes than other pathways, as exemplified by the interactions of this gene set with REACTOME_SIGNALING_BY_ERBB2 (Additional file 2). To better visualize the upstream/downstream relationships of these pathways, we constructed a directed weighted network of these pathways to demonstrate the interactions between these pathways (Fig. 2d). Negative values and values less than 1 were excluded before the construction of the adjacency matrix. This network clearly shows the upstream/downstream relationships of the oncogenic pathways and PID_P53_REGULATION_PATHWAY.

### Assembly of the enriched pathways based on GESTIA scores

Next, we demonstrate the ability of GESTIA to analyze the upstream/downstream relationships of enriched pathways in real datasets. We first analyzed the up/down-regulated pathways enriched by GSEA analysis of the RNA-seq data of TERT^high^ Vs. TERT^low^ hepatocytes from Lin et al. 2018 [19]. In this paper, GSEA revealed enriched gene sets associated with cell division and receptor tyrosine kinase activity in the TERT^High^ population, and enriched gene sets associated with ribosome components, mitochondrial proteins, the electron transport chain and hepatocyte metabolic activities in the TERT^Low^ population. However, the paper did not further explore the relationships between these enriched pathways.

**Fig. 3:**
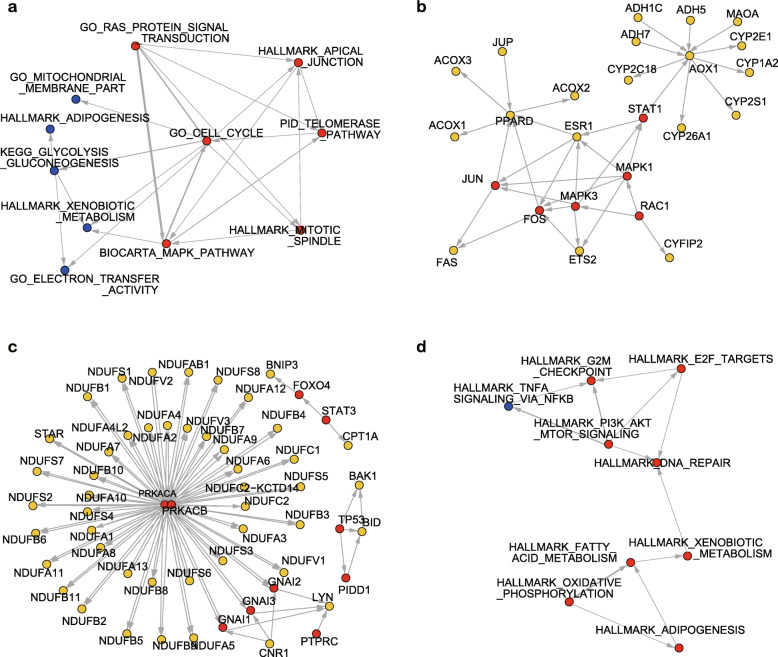
Assembly of enriched pathways from two real datasets based on GESTIA scores. **a.** The directed weighted network constructed using the GESTIA scores of the enriched pathways in Lin et al. 2018. Red vertices represent pathways that were up-regulated, and blue vertices represent down-regulated pathways. **b.** Visualization of the gene interactions between BIOCARTA_MAPK_PATHWAY and HALLMARK_XENOBIOTIC_METABOLISM (from MSigDB). Red vertices represent the genes in the first of the two pathways; the yellow ones represent genes in the second. To avoid overcomplicating the figure, we only showed the part of the figure where the two pathways interact with each other. **c.** Visualization of the gene interactions between GO_CELL_CYCLE and GO_MITOCHONDRIAL_ MEMBRANE_PART (from MSigDB). The color set of the vertices is the same as in b. **d.** The directed weighted network constructed using the GESTIA scores of the enriched pathways in Giustacchini et al. 2017. The color set of the vertices is the same as in a

After conversion of the GESTIA matrix (Additional file 6) to a directed weighted network (Fig. 3a), the assembly identified one super module consisted of two sub-graphs, with the GO_CELL_CYCLE functioning as a hub between them. We hereby define “GESTIA analysis” to refer to the whole processes of calculating GESTIA matrix, filtering and assembly of the super-module(s). Further demonstrations of the interactions between BIOCARTA_MAPK_PATHWAY and HALLMARK_ XENOBIOTIC_METABOLISM (Fig. 3b) and between GO_CELL_CYCLE and GO_MITOCHONDRIAL_MEMBRANE_PART (Fig. 3c) show that GESTIA analysis correctly detected the upstream/downstream relationships within these two pair of pathways. Notably, most of the genes in GO_MITOCHONDRIAL_MEMBRANE_PART do not form an interaction network, but GESTIA analysis still detects the relationships, which is mainly caused by the regulatory effects of Prkaca and Prkacb in GO_CELL_CYCLE on the Nduf gene family in GO_MITOCHONDRIAL_ MEMBRANE _PART (Fig. 3c). We further checked the source of the interactions between Prkaca/Prkacb and the Nduf gene family, and we found that Prkaca/Prkacb exhibits inhibitory effects on the Nduf genes in the Endogenous Cannabinoid Signaling Pathway (hsa04723). Although the mRNA level of Prkaca and Prkacb was not significantly changed, the genes Adcy6 and Adcy9, which catalyze the synthesis of cAMP, were up-regulated (adjusted *p*-value 0.049 and 0.095), increasing the activation of Prakaca and Prakacb, which would consequently inhibit the Nduf genes, consistent with the up-regulation of GO_CELL_CYCLE and the down-regulation of GO_MITOCHONDRIAL_MEMBRANE_ PART.

We then calculated the GESTIA matrix for the significantly enriched (adjusted *p*-value < 0.05) hallmark gene sets comparing remission BCR-ABL^−^ stem cells against remission BCR-ABL^+^ stem cells from another published work [20] and converted the GESTIA matrix (Additional file 7) to a weighted interaction network (Fig. 3d). Of the 13 enriched gene sets, 9 showed significant upstream/downstream relationships in a super module. The network clearly shows two distinct sub-networks: one is the signaling network of PI3K, AKT, mTOR, TNF-alpha, E2F and G2M check point; the other is the adipogenesis gene set and the network of oxidative phosphorylation, fatty acid and xenobiotic metabolism. The gene sets of G2M check point and DNA repair are the terminal vertices of the network, which is consistent with the elevated cell cycle/proliferation and DNA repair activities in remission BCR-ABL^−^ stem cells. Taken together, GESTIA analysis identified the upstream/downstream relationships of the enriched pathways and revealed the orchestrated regulation of pathways.

### Assembly of the refined pathways/modules based upon GESTIA scores

As revealed by Donato et al. 2013, unrelated pathways may gain significant p-value due to the overlapping genes with genuinely affected/causal pathways. The proposed “crosstalk analysis” can identify and filter these false positive and redundant pathways, at the same time create functional modules. Assembling the filtered pathways and newly created functional modules may generate a more concise and accurate map of how these pathways/modules interact with each other.

**Fig. 4:**
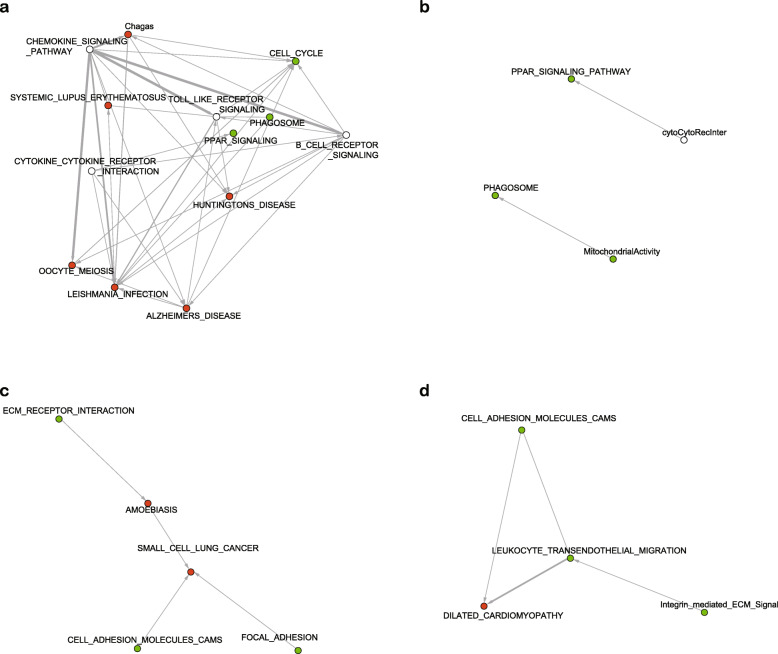
Assembly of pathways/modules with/without crosstalk analysis. **a.** Assembly of the enriched pathways from the fat remodelling study of Donato et al. 2013. **b.** Assembly of the pathways/modules after filtering by crosstalk analysis on the fat remodelling study. **c.** Assembly of the enriched pathways from the cervical ripening study of Hassen et al. 2009. **d.** Assembly of the pathways/modules from Hassen et al. 2009 after filtering by crosstalk analysis. Green vertices are pathways/modules that are known to be related to the study. Red vertices are obvious false positive pathways/modules. White vertices are uncertain pathways/modules

Since the examples presented in Donato et al. 2013 were well annotated with detailed explanation of the rationality of the results, we followed the works and applied GESTIA on both the unfiltered and filtered pathway analysis results. Figure 4a and b shows the assembled pathways and modules identified in the first example in Donato et al. 2013, which profiled the transcriptome of mice’s white fat tissue during transition into resembling brown fat tissue. The vertices in red are the pathways that were known to be unrelated to this biological process, while the green ones are supported by experimental evidences, and the blank ones are uncertain. Applying GESTIA analysis on the enriched pathways without crosstalk analysis keeps all of the three known related pathways, and excluded Parkinson’s Disease, Cardiac Muscle Contraction, Lysosome, and Complement and Coagulation Cascades, first two of which are known to be unrelated pathways (Fig. 4a). In Donato et al. 2013, crosstalk analysis shortened the list of the significantly enriched pathways and produced four additional functional modules. GESTIA analysis of these filtered pathways and functional modules shows there are at least two distinct super-modules in this context, involving the three known related pathways/modules and one previously uncertain module: Cytokine-Cytokine receptor interaction (Fig. 4b). The other two uncertain modules were left out in this assembly, which indicates that there were no significant upstream/downstream relationships between them and other pathways/modules. This is probably because the current pathway collections are still incomplete, hence there are unprofiled pathways which may link these two modules with other pathways/modules.

We also applied GESTIA analysis on the second example in Donato et al. 2013, which described cross talk analysis results of an experiment on cervical ripening. Figure 4c shows the GESTIA analysis result of the significantly enriched pathways without crosstalk analysis. The crosstalk analysis produced an independent functional module, extracted from the three pathways: ECM Receptor Interaction, Amoebiasis, and Focal Adhesion, named “Integrin Mediated ECM Signal” (Fig. 4d). Interestingly, the GESTIA analysis of the filtered pathways/modules (Fig. 4d) maintains the basic topological structure in Fig. 4c, with the replacement of the false positive pathway Small Cell Lung Cancer by Leukocyte Transendothelial Migration, and an additional downstream pathway Dilated Cardiomyopathy, which is labeled in red because of the lack of evidences. However, the strong GESTIA score between Dilated Cardiomyopathy and Leukocyte Transendothelial Migration led us to investigate the possibility of real involvement of this Dilated Cardiomyopathy pathway. We plotted the gene interactions between Dilated Cardiomyopathy pathway and Leukocyte Transendothelial Migration pathway (Additional file 8), which clearly showed three sub-modules in Dilated Cardiomyopathy pathway separated by Leukocyte Transendothelial Migration pathway. The three sub-modules correspond to 1. Sarcomere, 2. ECM-receptor interaction, and 3. Sarcoplasmic Reticulum derived calcium signaling. Each of these three sub-modules contains significantly changed genes, suggesting that they may all be involved in the process. The ECM-receptor interaction module has been detected by crosstalk analysis, as shown in Fig. 4d at the upstream of the super-module. The other two sub-modules are related to the muscle contraction, which was reasonable since 10 to 15% of the uterine cervix is constituted of smooth muscle [10].

## Discussion

Transcriptome profiling has spurred a move from functional analysis of single genes to pathways that contain multiple related genes. Numerous pathway analysis tools have been developed; these identify the most significantly impacted pathways by enrichment of orchestrated up/down regulation of genes in the pathway, providing stronger evidence for investigating the biological mechanisms than looking at the change of single gene’s expression level. Like individual genes, individual pathways also interact with each other, and the upstream/downstream pathways might be perturbed synergistically if there is an upstream change in the biological context. Therefore, it is possible that future pathway analysis will be able to interrogate the coordinated changes in multiple upstream/downstream pathways, assembling the orchestrated pathways into super functional modules and rendering better interpretations of the molecular mechanisms underlying the transcriptome profiles.

GESTIA score, as we present here, is able to quantify the upstream/downstream relationships of pathways/modules. Coordinately changed pathways/modules with upstream/downstream relationships will yield strong and significant GESTIA scores between each other, which enables the assembly of pathways/functional modules based on GESTIA scores. Other than breaking the pathways into sub-modules, GESTIA takes the gene interaction information to glue the pathways/modules together, which adds more rationality to the pathway/module analysis results and consequently helps to interrogate the mechanistic insights.

The input of GESTIA is two sets of genes. In theory, users can feed GESTIA with any gene sets, as long as they contain more than 5 genes. For instance, users can define their own pathways like “Mitochondria Activity”, then apply GESTIA on this user-defined pathway with other known pathways to assess the upstream/downstream relationships. The source of the input gene sets can be pre-defined gene sets (significantly enriched by pathway analysis, for instance), or gene sets constructed from co-expression analysis, or gene sets from module analysis from protein-protein interaction network, or any other methods that yield biological meaningful gene sets. This feature makes GESTIA highly flexible with input data. The output of GESTIA algorithm is a score estimating the upstream/downstream relationship between two pathways. The GESTIA scores can be arranged into a matrix, which can then be assembled into a directed graph. We call this directed graph “super-module” since it uses pathways as nodes. To distinguish the single GESTIA algorithm and the whole analysis starting from single GESTIA score calculation, matrix construction and super-module assembly, we call the later “GESTIA analysis”. The fundamental goal of GESTIA analysis is to elucidate the static upstream/downstream relationship between pathways, so that we can construct a weighted, directed pathway interaction network which is similar to gene interaction network despite that the nodes in the network are pathways.

The design of GESTIA score makes it favor the direct upstream/downstream relationships, especially the downstream genes in one pathway that directly affect the upstream genes in the other. The pathways that have intense interactions but do not exhibit such “tail to head” interactions will get low GESTIA scores. On the other hand, filtering by *p*-value will ensure that those strong GESTIA scores are generated from true upstream/downstream relationships, not by certain topological structures of the pathways (e.g. in pathway *A*, one gene controls ten genes; in pathway *B*, ten genes control one gene; if the interactions between *A* and *B* are random, then it is more likely to calculate a high positive raw GESTIA score for *A* over *B.* This kind of strong GESTIA score will ultimately be filtered by *p*-value).

We introduced pseudo vertices during the calculation of the null distribution, which may lack real biological meanings. However, the newly created pseudo vertices preserve the topological structures of both of the pathways, and at the same time, maintains the number of inter-pathway gene interactions (those arrows that start from one pathway and end in the other). We tested the raw influence score calculation on multiple pathway pairs with different topologies and with overlapping genes, with/without creating pseudo vertices. The raw influence scores remain the same with/without pseudo vertices, indicating that although the pseudo vertices did not serve as nodes with biological meanings, they did preserve the topological features of the two pathways. For the convenience of calculation, we decided to use this way of splitting the intersected gene sets in null distribution calculation.

Since the GESTIA score assesses the static pathway interactions, in theory we could apply GESTIA once and for all for every gene set of pathways, however, this would be too time consuming, although calculation of 10 to 20 input gene sets by GESTIA only takes a short period of time, which is sufficient for users to test GESTIA on their own interested pathways. Nevertheless, calculation of the GESTIA matrix of pathways from popular databases is still ongoing; the result for 179 KEGG pathways can be found at: https://github.com/yanshen2953/GESTIA/tree/master/PathwaysSetResult .

Since the GESTIA score calculation is based on a global network delineating all the possible gene interactions, the accuracy of this network may influence the result of the GESTIA score. With the accumulation and curation of pathway databases, this global network will be rapidly improved and, consequently, GESTIA will provide better estimation of the pathway relationships.

To generate a concise and accurate assembly of pathways/modules, another important factor is the accuracy of the pathway/module analysis itself. Take the crosstalk analysis result as an example, before filtering by crosstalk analysis, there are obvious false positive pathways in the list. These false positive pathways usually carry functional modules which grant the false positives upstream/downstream relationships with other genuine pathways with high possibility, resulting in inaccurate and redundant assembly of the pathways/modules. To date, there are numerous algorithms and tools to analyze pathway enrichment and generate functional modules, including construction of functional modules based upon protein-protein interaction networks or co-expression networks. GESTIA can be applied on top of these tools’ results and organize the pathways/modules into directed networks, which is helpful for the validation of the pathways/modules and the understanding of the molecular mechanisms.

For the weakness of GESTIA, firstly, the relationships between pathways are far more complex than just upstream/downstream relationships, but GESTIA can only assess a small sub-set of the pathway relationships (which is the upstream/downstream relationships). However, unlike the cooperative relationships (which can be assessed by extensively studied co-expression gene analysis) or functional relations (which can be assessed by algorithms like CSEA), the upstream/downstream relationships between pathways are far less quantitively studied (at least to our knowledge). Secondly, GESTIA analysis does not optimize the gene members of the pathway gene sets, like the crosstalk analysis proposed by Donato et al. 2013 do, nor does it construct new gene sets de novo from co-expression data or interactome data. Therefore, the quality of the super-modules produced by GESTIA analysis greatly relies on the quality of the input gene sets, i.e. whether the pathway gene set represents the real functional module in a certain study. Refinement of the pathway gene set can improve the quality of output of GESTIA analysis, as shown in Fig. 4. Thirdly, we currently only constructed the global gene interaction network for human. Assessment of the upstream/downstream relationships of pathways in other organisms may suffer from the inaccuracy of the gene interactions underlying the global network, which we do not recommend.

## Conclusions

GESTIA can be applied to a broad range of pathway/module analysis result, not just from transcriptome analysis, since the current GESTIA algorithm does not incorporate gene expression changes. It can assemble the candidate pathways/modules to form a topological structure, which reflects the internal biological relationships based on gene interactions. Additional tools to visualize such gene interactions between the pathways are also available in the R package that implements GESTIA. We hope that GESTIA may help researchers to get one additional step closer to understanding the molecular mechanism from the pathway/module analysis results.

## Methods

### Combining gene interactions into a global network

Due to the fact that genes often interact with other genes beyond a specific pathway, it is necessary to build a global network before assessing the interactions between pathways, otherwise the interactions of pathways will be limited to only the shared genes. To build a comprehensive gene interaction network, we combined 285 KEGG pathways. Currently, KEGG includes metabolic and non-metabolic pathways, which are totally different in expressing the correlations between genes. In metabolic pathways, the proteins (encoded by certain genes) often act as enzymes in biochemical reactions, so the substrates of the proteins are often metabolites. In contrast, in non-metabolic pathways, the proteins may directly modify the target proteins (phosphorylation, ubiquitination etc.), or bind to the target genes’ promoter/enhancer (as a transcription factor), then influence the expression of the target gene, or act as a epigenetic modifier that activates/suppresses the expression of certain genes. Due to these differences, some of the existing tools which built their own gene interaction networks excluded the metabolic pathways (e.g. CrossTalkZ, SPATIAL). In our study, we extracted the gene interactions of solely signalling pathways from KEGG by a R function, “KGML2igraph,” picked from the package “NetPathMiner”. We then converted the gene IDs into gene symbols and removed the isolated genes. The loops defined as “edges for which the two endpoints are the same vertex” within the igraph object was then removed using the function “simplify” from the R package “igraph”. Then, the igraph object was used as the global network in GESTIA calculation.

### Calculation of the raw GESTIA score

The purpose of the GESTIA score is to assess the upstream/downstream relationships of two pathways, which is essential to interrogate which of the two pathways has more of an impact on the other, and to what degree. Normally, if one gene can impact another gene (through activation, inhibition, induction, repression, etc.), we would conclude that the former is upstream of the later. Here, we extend this upstream/downstream notion from genes to pathways. For pathways, if the impact of the genes in pathway *A* on the genes of pathway *B* is more than the genes of *B* on *A*, we will conclude that *A* is upstream of *B*. Hence, we define the GESTIA score to be the normalized relative impact of pathway *A* on pathway *B*, so that if the normalized impact of *A* on *B* is higher than *B* on *A*, then GESTIA score will be positive, and vice versa. The input of GESTIA algorithm are two gene sets representing two pathways.

Next, we designed the algorithm to quantify the impact of one pathway on the other. Consider two pathways that do not have any overlapping genes but that do have interactions (e.g. Figure 1b). Since the genes in the two pathways reside in a comprehensive gene-gene interaction network, each gene in pathway *A* (e.g. red vertices in Fig. 1b) may or may not be directly upstream of the genes in pathway *B* (e.g. yellow vertices in Fig. 1b). Here we only consider the direct dominant effects of genes in *A*, for the indirect dominant effects will be accounted by the downstream genes in *A*. For example, in Fig. 1b, MAP 2 K2 does not directly impact IRS2, but its impact will be accounted in MAPK1’s impact. To achieve this adjustment of MAPK1’s impact, we assigned each gene with a weight to indicate its relative position in the network of the pathway. We then used this weight to adjust the influence scores of the downstream genes (e.g. MAPK1) to account for the indirect influence (e.g. the impact of MAP 2 K2). The weight of each gene in *A* is defined as the proportion of the downstream genes of *gene*_*i*_ in *A*:

$$ {w}_{Ai}=\frac{n_d}{n-1} $$

Where *w*_*Ai*_ is the weight of *gene*_*i*_ in pathway *A*, *n*_*d*_ is the number of genes that are downstream of *gene*_*i*_ in pathway *A*, and *n* is the number of genes in *A*. *n*-1 excludes *gene*_*i*_ in the count number. A similar definition can be applied to *w*_*Bi*_.

The weight we introduced for each gene is dependent on the relative position of the gene in the topology of the pathway. The downstream genes in the pathway will have smaller weights. The simplest scenario, in which pathway *A* is upstream of pathway *B*, would be that the downstream genes in *A* are directly upstream of the upstream genes in *B*. For example, if both *A* and *B* are linear, the ideal scenario is that the tail of *A* is upstream of the head of *B*. Therefore, we adjusted the influence score by timing 1-*w*_*Ai*_ to favor the downstream genes (e.g. MAPK1 in Fig. 1b), which also accounts for the impact of the upstream genes (e.g. MAP 2 K2 in Fig. 1b).

Currently, we do not have a comprehensive estimation of the strength of each gene’s impact; therefore, all of the impacts (edges in the network, e.g. Figure 1b’s edges) have a common weight which is 1. Hence, the impact of a gene in *A* (*Gene*_*Ai*_) on the whole pathway *B* is defined as the adjusted sum of its direct impact on *B*. For the same reason mentioned in the previous paragraph, we wanted to favour the “tail upstream of head” situation; therefore, the weights of genes in *B* (*w*_*Bi*_) were used to adjust each individual influence score before taking the sum, so that the genes impacted by *Gene*_*Ai*_ in *B* would be given higher weights if they were the “head” genes. Taken together, the influence score of *Gene*_*Ai*_ on *B* is given by the following formula:

$$ {InfluenceScore}_{Ai}=\left(1-{w}_{Ai}\right)\times \boldsymbol{DDs}\left({Gene}_{Ai},{Net}_{AB}\right)\bullet {\boldsymbol{w}}_B^T $$

Where *A* and *B* are the two gene sets for calculation, ***DDs*** stands for “**D**irect **D**own**s**tream,” which returns a 0-or-1 vector indicating whether the genes in *B* is directly downstream of *Gene*_*Ai*_. *Net*_*AB*_ is the network extracted from the global network based on the genes in *A* and *B*. *w*_*Ai*_ is the weight of *Gene*_*Ai*_*. w*_*B*_ is the vector of each gene’s weight in *B*. “T” means transpose. The dot product of ***DDs*** and transposed ***w***_*B*_ will give us the sum of the weights of *B*’s genes that are directly downstream of *Gene*_*Ai*_.

The *ImpactScore*_*Ai*_ basically takes the sum of the weights of genes in *B* that are directly downstream of *Gene*_*Ai*_, then uses the relative position of *Gene*_*Ai*_ to adjust the score. As suggested in the formula, we did not count the weights of the indirect downstream genes in *B*. If we include all the weights of the downstream genes in *B*, the information of the relative position of genes in *B* will be used more than once, which will introduce bias to favor the impact on those genes with higher weights in *B*.

The influence score of *A* on *B* is then defined as the sum of the influence score of each gene in *A* on *B*:

$$ {InfluenceScore}_A=\sum \limits_{i=1}^m\left(1-{w}_{Ai}\right)\times \boldsymbol{DDs}\left({Gene}_{Ai},{Net}_{AB}\right)\bullet {\boldsymbol{w}}_B^T $$

Where *m* is the number of genes in *A*.

The raw GESTIA score is then defined as:

$$ {GESTIA}_{A-B}={InfluenceScore}_A-{InfluenceScore}_B $$

$$ =\sum \limits_{i=1}^m\left(1-{w}_{Ai}\right)\times \boldsymbol{DDs}\left({Gene}_{Ai},{Net}_{AB}\right)\bullet {\boldsymbol{w}}_B^T-\sum \limits_{j=1}^n\left(1-{w}_{Bj}\right)\times \boldsymbol{DDs}\left({Gene}_{Bj},{Net}_{AB}\right)\bullet {\boldsymbol{w}}_A^T $$

We defined the raw GESTIA score of *A* on *B* as a subtraction of *B*’s influence score from *A*’s. This is based on the consideration of the complexity of two pathways’ interactions, where *A* may affect *B*, but at the same time, *B* may also affect *A*. In this situation, although *A* and *B* may intensively interact with each other, it is not certain which one of them acts the upstream of the other. Therefore, a subtraction of *B*’s influence score from *A*’s shows how much greater the strength of *A*’s impact on *B* is than that of *B* on *A*. At the same time, this definition also results in the symmetry of *GESTIA*_*A-B*_ and *GESTIA*_*B-A*_:

$$ raw\ {GESTIA}_{A-B}=- raw\ {GESTIA}_{B-A} $$

### Calculation of the empirical null distribution

Although the raw GESTIA score describes the relative impact of one pathway on the other, it is still possible that this impact is no more than the result of random effects. To remove the random effects, we first calculated the null distribution of the relative influence score of the two pathways, *A* and *B*. Since different pathways have different topological structures and different interactions between pathways, we applied random permutation on the edges that cross the pathways (starting from one pathway and ending at the other) by randomly select the starting genes and the ending genes while fixing the direction of the permutated edges to be the same with original one. For example, in a case wherein one edge originally starts from *A* and ends in *B*, the permutation was done by randomly selecting the starting gene from *A* and the ending gene from *B* (Fig. 1d). However, this permutation becomes difficult to implement when there are shared genes between the two pathways, since the change of the interactions related to the shared genes will also modify the topological structure of the pathways. We therefore created pseudo-vertices in the sub-network by duplicating the shared genes and their interactions, then split the sub-network into two non-overlapping pathways. (Fig. 1d). Then, we permutated the edges across the two newly formed pathways without disturbing the topological structures inside each pathway. Finally, we calculated the GESTIA scores of these randomly permutated sub-networks (*permImpactScore*_*A*_), which forms the empirical null distribution. The relative influence scores are then normalized by subtracting the mean of the permutated relative influence scores:

$$ normalized\ {InfluenceScore}_A={InfluenceScore}_A- mean\left({permInfluenceScore}_A\right) $$

The *p*-value was estimated by the chance of *permInfluenceScore*_*A*_ – *permInfluenceScore*_*B*_ to be more significant than *raw GESTIA*_*A-B*_. For pathways that do not interact at all, the GESTIA score will be 0 and *p*-value will be 1.

### Calculation of the normalized GESTIA score

We subsequently define the normalized GESTIA score to be:

$$ normalized\ {GESTIA}_{A-B}= normalized\ {InfluenceScore}_A- normalized\ {InfluenceScore}_B $$

Note that *permInfluenceScore*_*A*_ does not equal to *permInfluenceScore*_*B*_. This subtraction of two influence scores means that normalized GESTIA score is an estimation of the differences of the impact of pathway *A* on *B* and *B* on *A*.

## Supplementary information

**Additional file 1: Figure S1.** Visualization of the gene interactions between PID_ERBB2_ERBB3_PATHWAY and REACTOME_SIGNALING_BY_ERBB2. (DOCX 258 kb)

**Additional file 2: Figure S2.** Visualization of the gene interactions between REACTOME_PI3K_EVENTS_IN_ERBB2_SIGNALING and REACTOME_SIGNALING_BY_ERBB2. (DOCX 394 kb)

**Additional file 3.** R code for constructing global network. (TXT 2 kb)

**Additional file 4: Matrix Data 1.** JI matrix for Fig. 2a. (XLS 2 kb)

**Additional file 5: Matrix Data 2.** GESTIA score matrix for Fig. 2b. (XLS 3 kb)

**Additional file 6: Matrix Data 3.** GESTIA score matrix for Fig. 3a. (XLS 1 kb)

**Additional file 7: Matrix Data 4.** GESTIA score matrix for Fig. 3d. (XLS 5 kb)

**Additional file 8: Figure S3.** Visualization of the gene interactions between KEGG_DILATED_CARDIOMYOPATHY and KEGG_LEUKOCYTE_TRANSENDOTHELIAL_MIGRATION. (DOCX 741 kb)

**Additional file 9.** Additional discussion of the related issues. (DOCX 15 kb)

## Data Availability

All data generated or analysed during this study are included in this published article and its supplementary information files. The code generated in this study have been submitted to the Github (http://github.com/yanshen2953/GESTIA) under the afl-3.0 license.

## Abbreviations

GESTIA: GEne Set Topological Impact Analysis
ORA: Over Representative Analysis
FCS: Functional Class Scoring
PTB: Pathway Topology Based
GO: Gene Ontology
GSEA: Gene Set Enrichment Analysis
SPIA: Signaling Pathway Impact Analysis
KEGG: Kyoto Encyclopedia of Genes and Genomes
MSigDB: Molecular Signature DataBase
