# Supplementary material for: Analysing the meta-interaction between pathways by gene set topological impact analysis
Source: BMC Genomics. 2020 Oct 27;21:748. doi: 10.1186/s12864-020-07148-y (PMC7592530; doi:10.1186/s12864-020-07148-y)
Supplement: Supplementary file 1 — Additional file 1: Figure S1. Visualization of the gene interactions between PID_ERBB2_ERBB3_PATHWAY and REACTOME_SIGNALING_BY_ERBB2. (DOCX 258 kb) [file 12864_2020_7148_MOESM1_ESM.docx]

Figure S2. Visualization of the gene interactions between REACTOME_PI3K_EVENTS_IN_ERBB2_SIGNALING and REACTOME_SIGNALING_BY_ERBB2. Yellow vertices are genes in REACTOME_SIGNALING_BY_ERBB2, and green genes are shared genes.
