# Supplementary material for: Analysing the meta-interaction between pathways by gene set topological impact analysis
Source: BMC Genomics. 2020 Oct 27;21:748. doi: 10.1186/s12864-020-07148-y (PMC7592530; doi:10.1186/s12864-020-07148-y)
Supplement: Supplementary file 8 — Additional file 8: Figure S3. Visualization of the gene interactions between KEGG_DILATED_CARDIOMYOPATHY and KEGG_LEUKOCYTE_TRANSENDOTHELIAL_MIGRATION. (DOCX 741 kb) [file 12864_2020_7148_MOESM8_ESM.docx]

Figure S3. Visualization of the gene interactions between KEGG_DILATED_CARDIOMYOPATHY and KEGG_LEUKOCYTE_TRANSENDOTHELIAL_MIGRATION. Red vertices are genes in KEGG_LEUKOCYTE_TRANSENDOTHELIAL_MIGRATION, yellow vertices are genes in KEGG_DILATED_CARDIOMYOPATHY, and green vertices are shared genes. The squares are the significantly changed genes.
