# Supplementary material for: Analysing the meta-interaction between pathways by gene set topological impact analysis
Source: BMC Genomics. 2020 Oct 27;21:748. doi: 10.1186/s12864-020-07148-y (PMC7592530; doi:10.1186/s12864-020-07148-y)
Supplement: Supplementary file 9 — Additional file 9. Additional discussion of the related issues. (DOCX 15 kb) [file 12864_2020_7148_MOESM9_ESM.docx]

**Supplementary Discussion**

We have used the term “dominant effect” to express the notion that “pathway A controls pathway B”, like in this paper “PI3K-AKT-mTOR pathway is dominant over androgen receptor signaling in prostate cancer cells” (Kaarbø et al. 2010, Cell Oncol.). We want to measure this “dominant effect” by GESTIA. We started our algorithm design by observing the pathway interactions exemplified by cases in Fig. 1, which not only showed color-coded genes of different pathways, but also their directed interactions. The arrows in the figure showed the direction of the control between genes, e.g. MAP2K1 phosphorylate MAPK3 and activate its function, therefore MAP2K1 controls MAPK3’s function to a certain extent. We tried to extend this notion of “control” from gene interactions to pathway interactions, so that if most of the interactions between genes in pathway A and genes in pathway B is in the direction of A to B, we will conclude that pathway A “dominate” pathway B. Therefore, if two pathways (A and B) have many genes in common, we agree that they do have influence on each other, however, the direction of this influence can still vary. There are cases like Fig. 1a where the influences are bidirectional, therefore in our definition, it is hard to claim that pathway A controls/dominate pathway B or pathway B controls/dominate pathway A. On the other hand, in Fig. 1c, most of the arrows/influences start from REACTOME_PI3K_AKT_ACTIVATION and end in BIOCARTA_MTOR_PATHWAY, suggesting the former pathway controls/dominate the later. In summary, the “domination” between pathways in our definition means directed strong influence, which is not directly related to the number of common genes between pathways.

GESTIA captures the upstream/downstream relationships between pathways. The GESTIA value is a comparison of the degree of “domination” (influence score) between two pathways. For instance, assume that pathway A have strong influence on pathway B, which results in a big influence score, say 5.5, and at the same time, pathway B only have slight influences on pathway A, which results in an influence score of 0.5. Then we defined GESTIA score of A on B to be 5.5 – 0.5 = 5.0, suggesting that A’s influence of B is much stronger than the influence of B on A. This concept of “relative influence” was then converted to the “upstream/downstream relationships”, since we usually define an “upstream/downstream relationship” to be A controls B but B hardly affect A. (If B can also strongly affect A, then this relationship becomes a loop, not an upstream/downstream relationship).

Although higher GESTIA value indicates more distinct upstream/downstream relationships to some extent, it is still hard to compare the GESTIA values of two pairs of gene sets with different topologies, number of genes and number of inter-pathway gene interactions. For instances, if the GESTIA score of pathway A-B is 5 – 1 = 4, and the GESTIA score of pathway C-D is 100 – 90 = 10, although the later GESTIA score looks much higher than the former one, the former one’s upstream/downstream relationship is much more significant than the later one. Therefore, GESTIA algorithm also output a *p*-value, which estimates whether such GESTIA score is prominently higher (or lower if the values are negative) than the GESTIA scores calculated from randomized interactions. Considering the previous example, where it is likely that the randomized interactions will produce lots of GESTIA scores larger than 10 for the pathway pair C-D, the *p*-value won’t be significant, and we can filter this type of pathway pair out during the super-module assembly process.
